# Expanding the Clinical Spectrum of PIEZO2 Duplications: A Case of Global Motor Delay, Congenital Sensory Neuropathy, and Musculoskeletal Abnormalities

**DOI:** 10.7759/cureus.99113

**Published:** 2025-12-13

**Authors:** Grace Lee, Nicholas M Villar, Joseph Vo, Lin Kang

**Affiliations:** 1 Ophthalmology, Edward Via College of Osteopathic Medicine, Monroe, USA; 2 Orthopaedics, Edward Via College of Osteopathic Medicine, Monroe, USA; 3 Emergency Medicine, Edward Via College of Osteopathic Medicine, Monroe, USA; 4 Biomedical Sciences, Edward Via College of Osteopathic Medicine, Monroe, USA

**Keywords:** gene duplication, genetics, orthopedics, pediatrics, piezo2 gene

## Abstract

PIEZO2 encodes a mechanosensitive ion channel essential for touch perception, proprioception, and interoception, with biallelic loss-of-function variants known to cause a recessive mechanosensory neuropathy characterized by hypotonia and skeletal abnormalities; however, intragenic duplications have rarely been reported. We present a nine-year-old male with global motor delay, congenital hypotonia, distal muscle weakness, sensory neuropathy, thoracolumbar neuromuscular scoliosis, and multiple orthopedic abnormalities, along with microcephaly, cryptorchidism, chronic urinary incontinence, and early failure to thrive. Chromosomal microarray revealed a duplication at chromosome 18p11.22 involving PIEZO2, and exome-based copy number variant analysis confirmed a homozygous intragenic duplication. The duplicated exons are predicted to disrupt protein structure and function, resulting in impaired mechanotransduction, which is consistent with the patient’s proprioceptive deficits and musculoskeletal phenotype. This case likely expands to the mutational spectrum of recessive PIEZO2-related diseases. Care is supportive and multidisciplinary. Further case aggregation is needed to refine genotype-phenotype correlations.

## Introduction

Humans and other animals use mechanosensation, a process that includes the perception of touch, proprioception, and interoception to sense force and be able to interact with their environments [1]. However, how humans are able to perceive touch and proprioception is still relatively unclear, especially at the molecular level. In the last 10 years, PIEZO1 and PIEZO2 have been identified as having an essential role in mechanotransduction, a process that converts shear stress, pressure, or tension from outside forces into the electrochemical signals humans use to sense touch, proprioception, and interoception [2]. PIEZO1 and PIEZO2 are mechanically activated cation channels found in plants, protists, insects, vertebrate species, rodents, and humans. Both genes are expressed in the urinary bladder and lungs; however, PIEZO2 has been shown to be specifically expressed in dorsal root ganglia and in Merkel cells [1,2].

Mutations in the PIEZO2 gene can be responsible for many hereditary disorders. Loss-of-function mutations can lead to an autosomal recessive syndrome of muscular atrophy with perinatal respiratory distress, scoliosis, and arthrogryposis, a process described as multiple congenital contractures of the body [3]. Loss-of-function mutations lead to muscular atrophy of the lower limbs, kyphoscoliosis, and defective proprioception. Patients often present with arthrogryposis of the feet, wrists, or hands along with distal muscle weakness and atrophy [3]. In 2016, a study showed two unrelated patients with biallelic PIEZO2 loss-of-function mutations that presented with delayed walking, early impairment of fine motor skills, and delayed mastery of activities of daily living [1]. Neurological exam showed that both patients lost discriminative and light touch perception as well as proprioception. It was determined that the PIEZO2 gene is a major determinant of mechanosensation in humans and showed consistent results with studies on PIEZO2 knock-in mice [1].

Gain-of-function mutations can cause distal arthrogryposis type 3 (Gordon syndrome), distal arthrogryposis type 5, and Marden-Walker syndrome. Patients with gain-of-function mutations often present with generalized arthrogryposis, ophthalmoplegia and/or ptosis, and restrictive lung disease [1]. However, these mutations are limited in the literature and require further studies to better characterize the role of the mutation.

We present a case of an individual with a rare biallelic intragenic PIEZO2 gene duplication that has not been found in the literature. A thorough review and search throughout PubMed was conducted for similar mutations; however, only homozygous frameshift mutations leading to loss of PIEZO2 were described [4]. This case is notable due to its rarity and limited literature, as biallelic variants in PIEZO2 are not well described at this time, especially with the patient’s unique clinical presentation.

## Case presentation

A nine-year-old male was referred to our genetics clinic for evaluation and counseling in the setting of a complex medical and developmental history. His past medical history was notable for global motor developmental delay, generalized hypotonia, microcephaly, and short stature. He had a history of bilateral cryptorchidism treated with orchiopexy in early childhood, as well as thoracolumbar neuromuscular scoliosis currently under orthopedic monitoring. Neurologically, he demonstrated congenital sensory neuropathy and distal lower extremity weakness, which contributed to gait instability. Additional musculoskeletal findings included bilateral hallux valgus and pes planus. The patient had also experienced chronic urinary incontinence and had prior nutritional concerns, including failure to thrive requiring gastrostomy tube support during infancy. The patient and his parents have given written and verbal consent for reporting this case.

His birth history was significant for prematurity complicated by neonatal respiratory distress syndrome (NRDS), a patent foramen ovale (PFO), and multiple episodes of early-life bronchitis. Developmentally, he has continued to meet milestones at a delayed pace, requiring multidisciplinary outpatient therapies including physical, occupational, and speech therapy.

**Figure 1:**
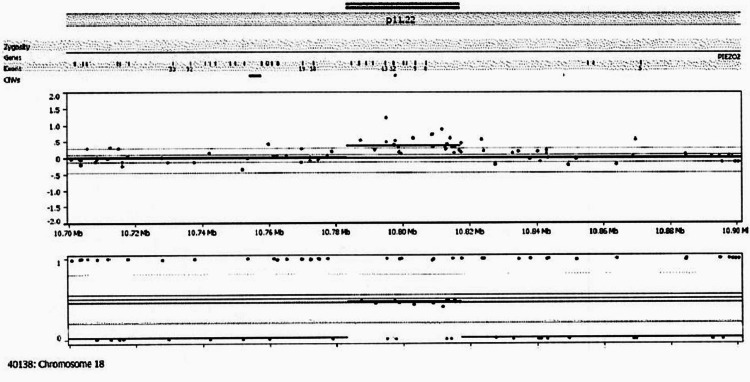
Chromosomal microarray results depicting male genomic profile with a variant of uncertain significance. ISCN: arr18p11.22(10,783,125-10,817,195)x3. Heterozygous copy number gain of 34 Kb on 18p11.22 seen on chromosomal microarray analysis. This region has not been reported to show copy number variation in the phenotypically normal population, nor has this specific duplication been associated with any known syndromes. This copy number gain is within the PIEZO2 gene (OMIM 613629); autosomal dominant distal arthrogryposis types 3 and 5 (DA3 and DA5; OMIM 114300 and 108145) are caused by a heterozygous mutation in the PIEZO2 gene. An autosomal recessive pattern of inheritance has also been postulated in some patients with an overlapping phenotype, Marden-Walker syndrome (OMIM 248700). ISCN: International System for Human Cytogenomic Nomenclature; OMIM: Online Mendelian Inheritance in Man.

Given the constellation of neuromuscular, skeletal, and sensory abnormalities, the patient underwent multi-tiered genetic testing. Initial chromosomal microarray (CMA) revealed a partial gene duplication involving exons 8-17 of the PIEZO2 gene, located at 18p11.22 with the coordinates (GRCh38): 10,666,483-11,149,569 (reverse strand). Subsequent Invitae 123 Neuromuscular Disorders Panel (Invitae, San Francisco, CA) identified three variants of uncertain significance (VUS) in three genes: COL6A3 (c.6342G>A), FLNC (c.4301G>A), and KBTBD13 (c.523C>T). All genes are associated with myopathic and myotonic phenotypes; however, these variants were not felt to fully explain the patient’s presentation. Unlike the VUS findings, the PIEZO2 gene has an established mechanistic link to these exact neurological and musculoskeletal features. Whole exome sequencing (WES) ultimately identified a pathogenic homozygous partial duplication of the PIEZO2 gene (Figure 1).

This partial duplication finding was consistent with the patient’s clinical features and was determined to be the primary molecular cause of his phenotype. While initial genomic testing identified several VUS in myopathy-associated genes, these variants did not clinically correlate with the patient’s neurological and musculoskeletal presentation. In contrast, the homozygous partial duplication of PIEZO2 detected on WES aligned closer with the patient’s constellation of congenital sensory neuropathy, hypotonia, motor developmental delay, skeletal anomalies, and genitourinary involvement. Given this strong phenotypic concordance and known mechanistic roles of PIEZO2 in proprioceptive signaling and musculoskeletal development, the duplication was determined to be the primary molecular etiology.

This case contributes to the growing characterization of PIEZO2-related disorders, which are known to involve mechanosensory dysfunction affecting proprioception, muscle tone, and skeletal development. Recognition of this pathogenic variant helped confirm the diagnosis, guide anticipatory management, and provide the family with both recurrence-risk counseling and targeted supportive care planning. Since parents were included in this testing, it was identified that they are each heterozygous for the partial duplication. The patient’s specific finding has not been seen in the literature; his features do seem consistent with autosomal recessive PIEZO2-related disorder, such as scoliosis, short stature, neonatal hypotonia, and muscle weakness. Arthrogryposis can also be seen, but at this time, he does not appear to have joint contractures. It is hard to say what this patient’s future looks like, so his clinical picture and molecular findings will need to continue to be followed.

At present, there is no definitive cure or disease-modifying therapy for disorders associated with PIEZO2 gene duplication. Management is supportive and focuses on optimizing function, monitoring progression, and addressing individual symptoms through multidisciplinary care. The patient has been referred to pediatric orthopedics for ongoing evaluation and management of his thoracolumbar neuromuscular scoliosis and associated gait abnormalities. Pediatric neurology continues to follow him for assessment of congenital sensory neuropathy and distal muscle weakness. Due to persistent concerns regarding growth delay and limited caloric intake, appetite stimulants have been initiated, with nutritional services and endocrinology consulted for ongoing support and monitoring.

Overall, care remains centered on maximizing functional independence, monitoring musculoskeletal progression, and offering coordinated multidisciplinary support to address the complex sequelae of this rare PIEZO2-related neuromuscular phenotype.

## Discussion

Biallelic pathogenic variants in the PIEZO2 gene cause a recessive neuromuscular syndrome characterized by impaired proprioception, muscular atrophy, hypotonia, depressed or absent deep tendon reflexes, delayed motor milestones, scoliosis, contractures, and foot deformities such as talipes equinovarus. Arthrogryposis is frequently observed, but phenotypic variability is well-documented, and some patients may lack certain features, such as arthrogryposis or have isolated contractures, as seen in this case. Intellect is typically preserved in affected individuals, and there is no evidence for cognitive impairment or central nervous system involvement on imaging or neurophysiological studies [1,5,6].

The pathophysiology is attributed to loss of PIEZO2-mediated mechanotransduction in the dorsal root ganglia and proprioceptive sensory neurons, leading to abnormal muscle development and joint positioning. This mechanism is supported by both human and animal studies, which demonstrate that PIEZO2 deficiency disrupts proprioceptive feedback and skeletal integrity [7,8].

Autosomal dominant PIEZO2 variants are associated with distinct phenotypes, including distal arthrogryposis types 3 and 5 and Marden-Walker syndrome, which do not match the recessive presentation [1]. Genotype-phenotype correlations remain limited due to the rarity and diversity of reported mutations, and intragenic duplications have not been previously described.

Features such as microcephaly and urinary incontinence are not well-established components of the PIEZO2-related disorder spectrum. The overall prognosis is unclear due to limited longitudinal data, but the disorder is generally slowly progressive and not life-threatening [1,6].

Recurrence risk for future offspring of the parents is 25%, consistent with autosomal recessive inheritance, but the precise risk depends on carrier frequency in the population [9]. Variants in COL6A3, FLNC, and KBTBD13 are likely not contributory to the phenotype, as supported by segregation analysis [2]. Genetic counselors play a critical role in helping families understand and manage genetic diagnoses, particularly in the context of reproductive decision-making. Achieving a genetic diagnosis enables families to consider options such as preimplantation genetic testing (PGT), which can reduce the likelihood of having affected offspring if the parents are known carriers. The American Society for Reproductive Medicine specifically recommends that any patient considering PGT for monogenic conditions should receive consultation from a genetic counselor, who can provide detailed information about the natural history of the condition, genotype-phenotype correlations, and the feasibility of PGT, as well as other reproductive options. Genetic counseling is especially important in populations with high rates of consanguinity [10].

Currently, there is no specific cure for PIEZO2 gene disorders, and treatment options are largely symptom-specific with a multidisciplinary team. One newer study has shown that linoleic acid improves PIEZO2 dysfunction in a mouse model of Angelman syndrome and is a potential route for further research [11]. Considering the rarity of this case, further correlation with other similar cases is necessary, as well as additional research into potential treatment modalities.

## Conclusions

This case represents a previously unreported instance of a biallelic intragenic duplication of the PIEZO2 gene presenting as a recessive neuromuscular and proprioceptive disorder. This case provides new insight into a limited spectrum of PIEZO2-related disorders. Although the patient presented with symptoms that align with previous literature, he also showed atypical findings like microcephaly and urinary incontinence. As the current treatment of this disorder is supportive, continued documentation and research into potential therapies should be explored to improve clinical care for affected individuals.
